# Case Report: Recurrent vulvar abscess as the initial manifestation of Crohn’s disease in an adolescent

**DOI:** 10.3389/fmed.2026.1763956

**Published:** 2026-01-29

**Authors:** Ming He, Huimin Ma, Na Huo, Junxia Li, Xiangyan Li

**Affiliations:** 1 Department of Infectious Diseases, Peking University First Hospital, Peking University, Beijing, China; 2 Department of Gastroenterology, Peking University First Hospital, Peking University, Beijing, China

**Keywords:** adolescent, Crohn’s disease, fecal calprotectin, ustekinumab, vulvar abscess

## Abstract

**Introduction:**

Vulvar abscess as the primary and sole manifestation of Crohn’s disease (CD) is extremely rare, particularly in adolescents lacking typical gastrointestinal symptoms. This atypical presentation frequently leads to misdiagnosis and delayed treatment.

**Case description:**

We report the case of a 16-year-old female admitted for recurrent vulvar abscesses accompanied by high fever. Initial management involved local drainage and antibiotics, but efficacy was limited. Upon admission, targeted history taking revealed a change in bowel habits. Laboratory results indicated a significant inflammatory response and positive fecal calprotectin. Combined with imaging, endoscopy, and pathology, the patient was diagnosed with ileal CD. A multidisciplinary treatment strategy was adopted. This included standardized drainage, necessary antibiotic coverage, and exclusive enteral nutrition (EEN) combined with ustekinumab to achieve steroid-free induction. The patient achieved both clinical and mucosal remission.

**Conclusion:**

This case highlights that recurrent vulvar abscesses in adolescents warrant a high index of suspicion for CD. Clinicians must prioritize targeted history taking. Furthermore, fecal calprotectin testing and imaging should be combined to identify potential inflammatory bowel disease (IBD) early, thereby avoiding missed diagnoses and treatment delays.

## 1 Introduction

Crohn’s disease (CD) is a chronic inflammatory bowel disease (IBD) in which vulvar involvement represents a rare and clinically challenging extraintestinal manifestation. These lesions are generally classified into two categories: contiguous lesions extending directly from anorectal disease, and non-contiguous “metastatic” granulomatous lesions separated from the gastrointestinal tract (1). Because vulvar signs may precede gastrointestinal symptoms or overshadow mild intestinal manifestations, misdiagnosis is frequent. We report a case of adolescent CD initially presenting as recurrent vulvar abscesses accompanied by persistent high fever. Physical examination revealed local tenderness, a residual drainage opening, and purulent discharge. Laboratory investigations indicated a severe systemic inflammatory response, notably a white blood cell count of 21.3 × 10^9^/L, C-reactive protein of 71.4 mg/L, and an erythrocyte sedimentation rate of 92 mm/h. This article highlights the importance of in-depth history taking, non-invasive screening, and clinical reasoning to achieve early diagnosis in the absence of typical digestive symptoms. Furthermore, it discusses the selection of appropriate biologic therapies for moderate-to-severe CD complicated by secondary infection.

## 2 Case presentation

### 2.1 General information and history

A 16-year-old female was admitted due to “intermittent high fever (maximum T 39.7 °C) accompanied by painful swelling of the left vulva for 28 days.” She was previously healthy. Prior to admission, she was diagnosed with a “vulvar abscess” at another hospital. Despite incision, drainage, and multiple courses of broad-spectrum antibiotics, symptoms recurred and progressively worsened. Initially, the patient did not volunteer any gastrointestinal complaints. However, during targeted history taking after admission, she reported a change in bowel habits over the past 6 months. This manifested as soft, unformed stools 1–2 times daily, with occasional watery stools. Although there was no obvious abdominal pain, hematochezia, or weight loss, this “occult symptom” became the diagnostic breakthrough.

### 2.2 Physical and auxiliary examinations

**Figure 1:**
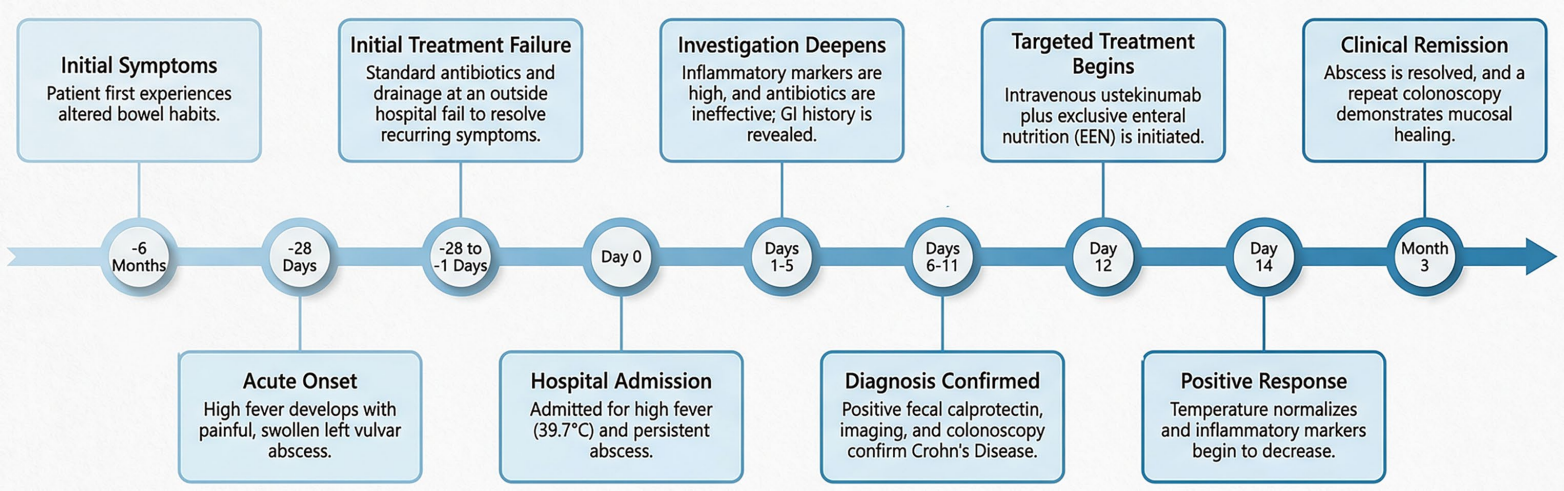
Timeline of the case. GI, Gastrointestinal.

Physical examination revealed tenderness in the left vulva, with a residual drainage opening and a small amount of purulent discharge. Laboratory tests indicated a severe systemic inflammatory response: white blood cell count 21.3 × 10^9^/L, C-reactive protein (CRP) 71.4 mg/L, and erythrocyte sedimentation rate (ESR) 92 mm/h. Despite prior broad-spectrum antibiotic treatment, inflammatory markers remained elevated (Figure 1).

### 2.3 Diagnostic clues and confirmation process

Pathogen Detection and Analysis: Bacterial culture and tNGS (targeted next-generation sequencing) of the pus identified *Klebsiella* var*iicola* and *Veillonella parvula*. However, even after escalating treatment to potent antibiotics like meropenem, the high fever and abscess remained uncontrolled. Given the poor response to conventional therapy, further screening for systemic inflammation was initiated.

Specific markers: The fecal calprotectin (FC) qualitative test was positive, suggesting active intestinal inflammation.

**Figure 2:**
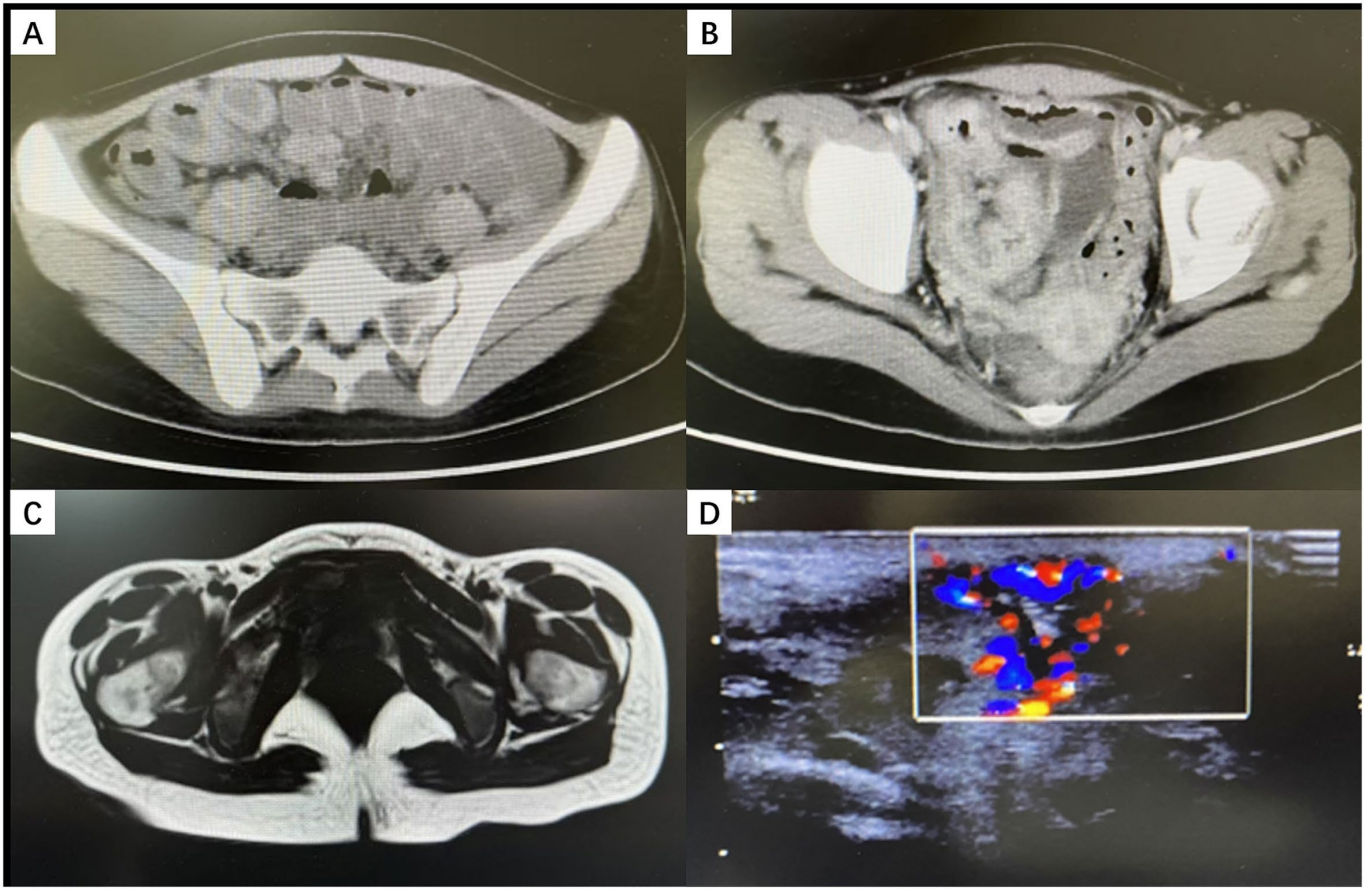
Imaging findings **(A,B)** Contrast-enhanced abdominal CT showing marked thickening and enhancement of the terminal ileum. **(C)** Contrast-enhanced pelvic MRI showing post–incision and drainage changes of a left vulvar abscess, with an abnormal signal posterior to the left vaginal introitus, suggesting a residual abscess. **(D)** Perineal ultrasonography revealing a left-sided perineal abscess with abundant blood flow.

Imaging: Contrast-enhanced abdominal CT showed segmental thickening of the terminal ileum and adjacent bowel segments, accompanied by mesenteric lymphadenopathy (Figures 2A,B). Perineal ultrasound and pelvic MRI indicated a subcutaneous abscess cavity in the left vulva extending toward the anal canal and rectovaginal septum (Figures 2C,D). While the full fistula track was not clearly delineated, a potential fistula was suspected.

**Figure 3:**
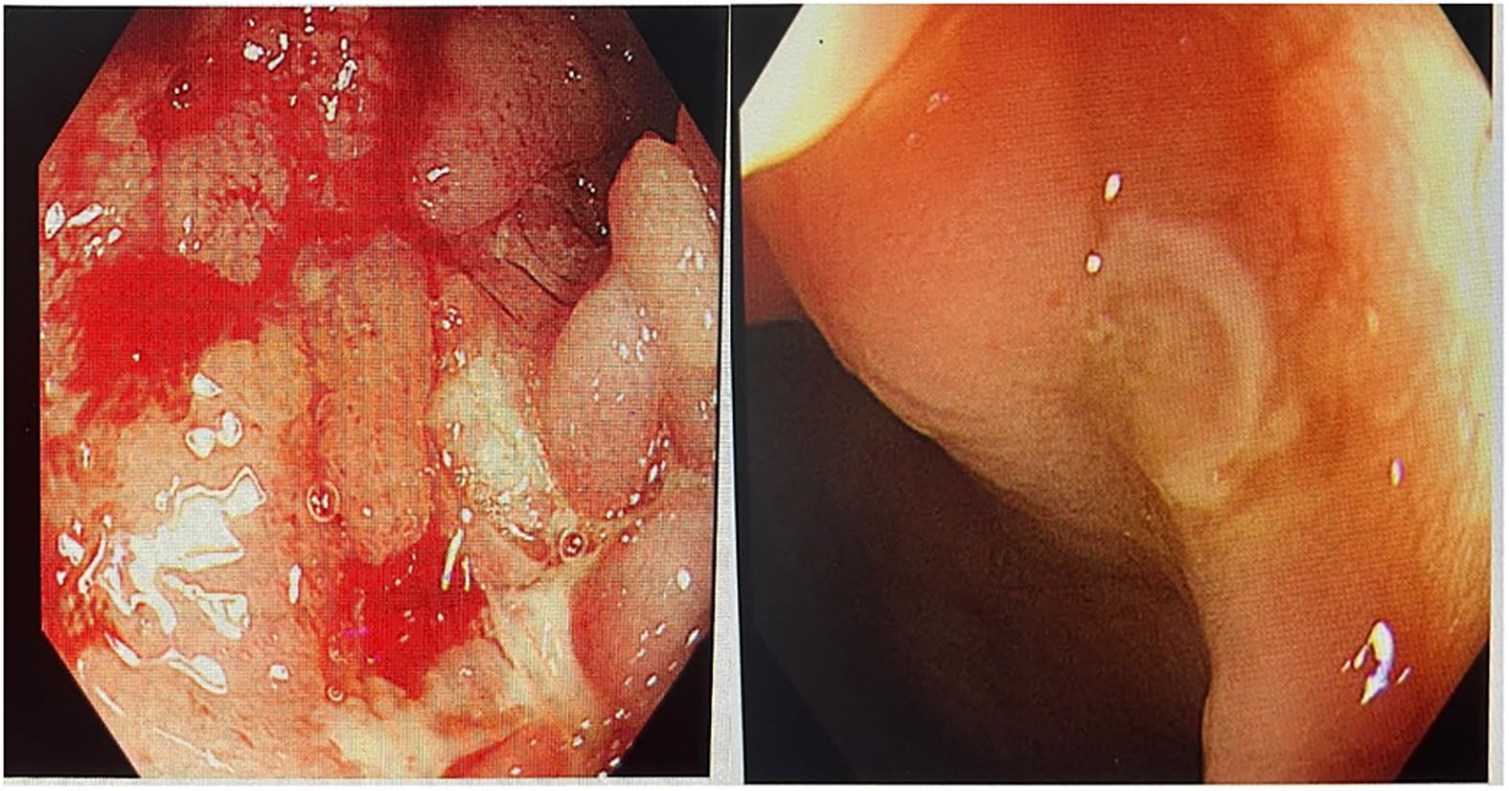
Colonoscopy showing longitudinal ulcers in the ileocecal region.

Endoscopy and pathology: Colonoscopy revealed multiple longitudinal ulcers and typical “cobblestone” changes in the terminal ileum (Figure 3). Histopathology showed chronic active inflammation with non-caseating granuloma formation.

Based on these findings, the patient was diagnosed with CD (Montreal classification: A1 L1 B1p, moderate activity).

### 2.4 Treatment and clinical course

A multidisciplinary team (MDT) formulated the following plan: Anti-infection and Local Management: Local debridement and drainage were performed. Meropenem was continued based on susceptibility results to control secondary bacterial infection. Nutritional Support: Exclusive enteral nutrition (EEN) was initiated to improve nutritional status and assist in inducing remission. Biologic Induction: Given the local infection and the need for a favorable immune environment for vulvar healing, systemic corticosteroids were avoided. Ustekinumab (UST), which has a favorable safety profile, was selected. An initial intravenous induction dose of 260 mg was administered, followed by a second induction dose of the same quantity 2 weeks later, before transitioning to maintenance therapy. Following the initiation of UST, the patient’s temperature rapidly normalized, and inflammatory markers gradually returned to normal ranges. At the 3-month follow-up, abdominal CT showed significant resolution of small bowel inflammation, and the vulvar abscess had completely resolved. Repeat colonoscopy demonstrated mucosal healing in the ileum, with only scattered superficial erosions remaining. During the follow-up period, the patient reported formed stools and no recurrence of perineal discomfort, achieving both clinical and endoscopic remission.

## 3 Discussion

This case highlights the diagnostic pitfalls of adolescent CD presenting with recurrent vulvar abscesses. In previous literature, CD-related vulvar lesions typically present as edema, ulcers, and erythema; onset as an “abscess/sepsis-like presentation” is extremely rare (2). We identified six similar cases in the literature where vulvar abscess was the initial presentation of CD and compared them with the current case (Table 1).

**Table 1:** Clinical characteristics of reported cases of CD initially presenting with vulvar abscesses (all cases were female).

Case	Age (year)	Country	Time to diagnosis	GI symptoms	Fever	Anal fistula	Diagnostic methods	Treatment
1 (8)	22	UK	3 years	Yes	No	Yes	Colonoscopy + pathology	Abscess ostomy (CD treatment unknown)
2 (9)	18	Korea	5 months	No	No	Yes	Imaging, colonoscopy + pathology	Antibiotics, incision and drainage (I&D); elemental diet + azathioprine + mesalazine; fistulectomy
3 (10)	12	United States	2 years	Yes	Transient, low-grade	No	Vulvar biopsy, colonoscopy + pathology	Prednisone
4 (11)	14	United States	16 months	Yes	No	Yes	Imaging, colonoscopy + pathology	Antibiotics, I&D; infliximab
5 (12)	10	United States	5 years	No	No	Yes	Colonoscopy + pathology	I&D; prednisone, colchicine, methotrexate, azathioprine, infliximab; fistulectomy
6 (13)	28	Canada	9 months	Yes	No	No	Vulvar biopsy, surgical bowel resection pathology	I&D; bowel resection
7 (Current)	16	China	1 month	No	High fever	Yes	FC, imaging, colonoscopy + pathology	Antibiotics, I&D, EEN, ustekinumab

CD, Crohn’s disease; GI, gastrointestinal; I&D, incision and drainage; EEN, exclusive enteral nutrition.

Through literature review and analysis of this case, we summarize the following key clinical insights:

### 3.1 Diagnostic pitfalls and coping strategies: valuing “occult clues”

Vulvar abscesses are often viewed as local infections. However, when they recur, involve deep spaces, or respond poorly to drainage/antibiotics, CD should be included as a major differential diagnosis. A review of the six similar cases (Table 1) reveals a long average delay in diagnosis. The success in the current case is attributed to:

#### 3.1.1 Targeted history taking

Even without spontaneous complaints, the physician actively inquired about symptoms, uncovering changes in bowel habits. This prompted a gastrointestinal assessment and shortened the time to diagnosis. In clinical practice, we recommend routine inquiry for patients with recurrent vulvar abscesses regarding: bowel habit changes, abdominal pain/distention, perianal symptoms (discharge, pain), oral ulcers, skin/joint symptoms, and growth development. It is important to note that in adolescents, distinct weight loss may not always be evident; instead, growth stagnation or inadequate weight gain can be a subtle but equivalent indicator of chronic disease.

#### 3.1.2 The value of FC and imaging

Guidelines recommend FC to differentiate inflammatory from non-inflammatory bowel diseases (3). As a non-invasive, sensitive marker of intestinal inflammation, FC serves as an effective screening tool for vulvar abscesses unresponsive to antibiotic treatment to identify potential IBD. Regarding imaging, pelvic contrast-enhanced MRI is superior for assessing deep perineal spaces, rectovaginal septum involvement, and potential fistulas. Therefore, we recommend early pelvic MRI for recurrent or persistent vulvar abscesses to rule out deep undrained cavities. Abdominal contrast-enhanced CT can reveal small bowel lesions, such as those in the terminal ileum. In this case, a positive FC screen combined with imaging findings provided a critical chain of evidence, leading to colonoscopy and pathological confirmation.

#### 3.1.3 Differential diagnosis and multidisciplinary strategy

Given the atypical presentation, differentiating vulvar CD from other etiologies is critical. Initially, this case required differentiation from Bartholin’s abscess, vulvar cellulitis, hidradenitis suppurativa, vulvar tuberculosis, sexually transmitted diseases, and Behçet’s disease. These were effectively excluded based on the lack of response to conventional anti-infective therapy, positive fecal calprotectin screening, and specific endoscopic findings. A multidisciplinary approach involving infectious disease specialists, gastroenterologists, gynecologists, surgeons, and dermatologists is essential for such complex cases. Regarding histopathological confirmation, although a vulvar biopsy is valuable for definitive diagnosis, it was deferred in this patient. The decision was made to avoid invasive procedures on the highly inflamed and edematous tissue, which could lead to iatrogenic fistulas or non-healing wounds. Instead, the diagnosis was successfully established through the combination of ileal pathology and the exclusion of other etiologies.

### 3.2 Literature review: clinical spectrum

Comparing this case with the six identified in the literature (Table 1), the mean age of onset was 17.3 years (range: 10–28 years), similar to our patient. This suggests a need for heightened vigilance in the adolescent population. Furthermore, previous cases mostly presented with a chronic, lingering course without fever (characteristic of “cold abscesses”). In contrast, our patient presented with high fever (39.7 °C) and a severe systemic inflammatory response. This indicates that the clinical spectrum of vulvar CD is broad; it can present not only as chronic granulomas but also as acute, sepsis-like changes with high inflammatory burden.

### 3.3 Treatment strategy: biologic selection in high-infection risk settings

Currently, guidelines strongly recommend anti-TNF-α monoclonal antibodies and interleukin inhibitors (e.g., UST) as first-line therapies for induction and maintenance in moderate-to-severe CD (3, 4). In pediatric and adolescent guidelines, anti-TNF drugs are often preferred due to stronger evidence (5). In the similar cases we reviewed that required biologic therapy, infliximab was the agent used. While infliximab is extensively documented, other anti-TNF agents such as adalimumab have also demonstrated efficacy in vulvar CD (6). However, the choice of biologic in this case was heavily influenced by the concurrent severe infection. In the acute phase accompanied by abscess and high fever, the use of anti-TNF agents, systemic steroids, or immunosuppressants carries a risk of exacerbating infection dissemination.

This case utilized a regimen of UST combined with EEN. UST precisely inhibits inflammation by blocking the IL-12/23 pathway and is associated with a relatively lower risk of serious opportunistic infections (7). When combined with the nutritional support, anti-inflammatory effects, and mucosal repair properties of EEN (5), this approach successfully achieved “steroid-free” induction of remission. This strategy not only avoided the infection risks associated with broad immunosuppression but also achieved dual healing of the vulvar lesion and intestinal mucosa, confirming its feasibility in complex adolescent CD cases.

## 4 Conclusion

When adolescent females present with recurrent vulvar abscesses—especially those accompanied by systemic inflammatory reactions disproportionate to local signs and poor response to conventional drainage/antibiotics—CD should be highly suspected. Clinicians must prioritize targeted history taking and combine fecal calprotectin screening with pelvic/abdominal imaging to facilitate early endoscopic and pathological confirmation. Regarding treatment, alongside standardized local management and necessary antibiotic coverage, timely initiation of induction therapy targeting CD activity (such as a steroid-free regimen of UST combined with EEN) contributes to the synchronous control and long-term remission of both vulvar lesions and intestinal inflammation.

## 5 Patient perspective

“I suffered from the painful swelling and high fever for a month, and the multiple surgeries and antibiotics did not help, which was terrifying. I never thought my mild stomach issues were related. I am grateful the doctors asked detailed questions about my digestion. After starting the new medication and the nutrition drink, my fever stopped quickly, and the pain finally disappeared. I am now back to school and living a normal life.”

## Data Availability

The original contributions presented in the study are included in the article/supplementary material, further inquiries can be directed to the corresponding author.
